# Simultaneous Determination of Structurally Diverse Compounds in Different Fangchi Species by UHPLC-DAD and UHPLC-ESI-MS/MS

**DOI:** 10.3390/molecules18055235

**Published:** 2013-05-07

**Authors:** Hee-Jung Sim, Ji Hee Kim, Kang Ro Lee, Jongki Hong

**Affiliations:** 1 College of Pharmacy, Kyung Hee University, Hoegi-dong, Dongdaemoon-gu, Seoul 130-701, Korea; E-Mails: hjsim@kriss.re.kr (H.-J.S.); jhhhw23024@hanmail.net (J.H.K.); 2 Division of Metrology for Quality of Life, Korea Research Institute of Standards and Science, Yuseong, Daejeon 305-600, Korea; 3 College of Pharmacy, Sungkyunkwan University, 300 Chonchon-dong, Jangan-ku, Suwon 440-746, Korea; E-Mail: krlee@skku.ac.kr

**Keywords:** Fangchi species, alkaloids, aristolochic acid I, lignan, UHPLC-DAD, UHPLC-MS/MS

## Abstract

Two bisbenzylisoquinoline alkaloids, two morphine alkaloids, one aporphine alkaloid, syringaresinol and aristolochic acid І were selected as marker compounds and simultaneously analyzed using an ultra-high pressure liquid chromatography-diode array detection (UHPLC-DAD) method. These marker compounds were used for the quality control of Fangchi species of different origins, including *Sinomenium* *acutum*, *Stephaniatetrandra*, *Cocculus* *trilobus* and *Aristolochia* *fangchi*. A reversed-phase UHPLC-DAD method was developed and validated for the simultaneous quantification of structurally diverse markers in different Fangchi species. In addition, an UHPLC-electrospray ionization tandem mass spectrometry (ESI-MS/MS) method was used for marker identification in Fangchi species, which provided diagnostic MS/MS spectral patterns that were dependent upon the marker structures. The UHPLC-MS/MS data were used to confirm and complement the UHPLC-DAD quality evaluation results. Additionally, magnoflorine and syringaresinol were observed for the first time in *S. tetrandra* and *C. trilobus*, respectively. Twenty different Fangchi species samples were analyzed for aristolochic acid I, syringaresinol and the alkaloids using the UHPLC-DAD and MS/MS method. Based on the levels of markers and principal component analysis (PCA), this method allowed for the clear classification of the samples into four different groups representing samples originating from the four species.

## 1. Introduction

Fangchi, one of the most commonly used traditional herbal medicines, is derived from the rhizoma of *Sinomenium (S.) acutum* [1,2] and the radix of *Stephania* (*S.) tetrandra* (Menispermaceae) [3]. *S. acutum* and *S. tetrandra* have been widely used for the treatment of rheumatic arthritis [4]. The main bioactive components in *S. acutum* are alkaloids and lignans such as sinomenine, isosinomenine, magnoflorine and syringaresinol [5]. The main bioactive components in *S. tetrandra* are tetrandrine and fangchinoline [5]. Additionally, *Cocculus* *(C.) trilobus* (Menispermaceae) and *Aristolochia* *(A.) fangchi* (Aristolochiaceae) are also referred to as “Mu fangchi” and “Guang fangchi”, respectively. *C. trilobus* has been used in folk medicine as a diuretic, analgesic and an anti-inflammatory [6]. *A. fangchi* has been banned for medicinal use because of the presence of aristolochic acids, which are known nephrotoxins and carcinogens [7]. The chemical compositions of Fangchi extracts differ significantly in terms of the species from which they originate. Furthermore, the biological activity of each Fangchi species is closely related to its major chemical components.

Aporphine, morphine and bisbenzylisoquinoline alkaloids, lignans and nitrophenanthrene carboxylic acids are the major components of Fangchi species and have various pharmaceutical effects. It was reported that magnoflorine, an aporphine alkaloid, has antioxidant activity by protecting human high-density lipoprotein against lipid peroxidation [8]. Morphine alkaloids, such as sinomenine and isosinomenine, have been reported to have anti-angiogenic, anti-inflammatory and anti-rheumatic effects [9]. The bisbenzylisoquinoline alkaloids fangchinoline and tetrandrine have shown anti-inflammatory, hypotensive and vasodilating effects [10,11]. Syringaresinol, a furofuran-type lignan, has been shown to inhibit the motility of *Helicobacter pylori* [12] and to possess anti-proliferative activity [13]. Aristolochic acid I caused rapidly progressive interstitial nephritis in patients who ingested slimming pills derived from herbal medicines containing aristolochic acid I [14]. 

However, analytical methods have only been narrowly applied for quality evaluations of members of this genus due to the challenge of detecting structurally diverse compounds that vary with Fangchi origin. Moreover, the identification and determination of the bioactive compounds in herbal medicine is a difficult process because it necessitates LC chromatographic separation of structurally diverse compounds and can lead to false positives due to overlapping interferences or structurally similar components present in complicated herbal extracts. Thus, the development of a practical UHPLC method for the identification and simultaneous determination of structurally diverse markers is essential for quality evaluations of Fangchi species of different origins. The UHPLC method had advantages over HPLC in terms of time saving, solvent saving, performance, and efficiency that resulted in higher sample throughput, less solvent consumption, and less sample injection volume than HPLC.

**Figure 1:**
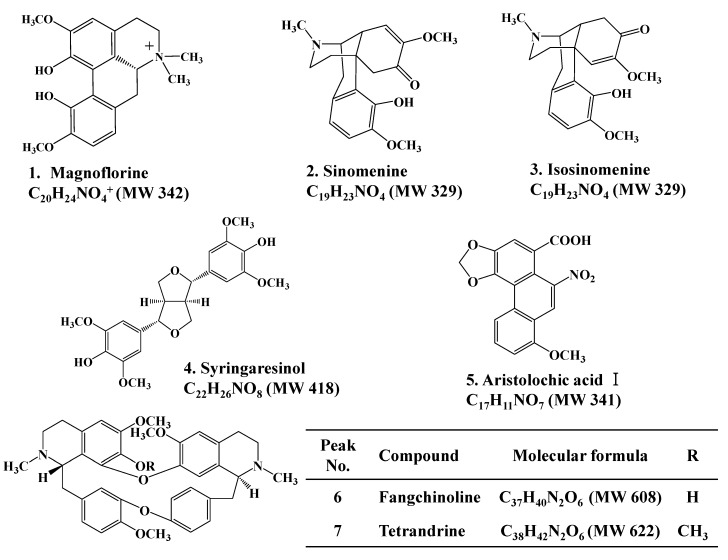
Chemical structures of compounds identified in four Fangchi species.

This study demonstrated a rapid and simple UHPLC-DAD method for the simultaneous determination of structurally diverse markers (as shown in Figure 1) in Fangchi species of different origins. The validated UHPLC method was successfully applied for the simultaneous determination of these marker compounds in twenty Fangchi samples. All Fangchi samples were also analyzed using an UHPLC-ESI-MS/MS method to confirm the UHPLC results. The optimized UHPLC methods were used to determine the levels of these marker compounds in twenty Fangchi extracts and to classify the samples into four different Fangchi species through PCA. This study represents the first investigation towards the simultaneous determination of structurally diverse marker compounds in four Fangchi species of different origins. The described methods are applicable for identification and analytical profiling of the four Fangchi species. Even more, they can be used to recognize falsifications and for quality control of medicinal preparations containing Fangchi herbs.

## 2. Results and Discussion

### 2.1. Analysis of Marker Compounds by UHPLC-DAD

UHPLC separation conditions were optimized to facilitate the simultaneous determination of various markers (alkaloids, aristolochic acid I and a lignan) in Fangchi species in a single LC run. The optimized parameters were pH, NH_4_OAc concentration and the composition of the mobile phase, as performed in our previous report [15]. In addition, the retention of ionizable compounds using reversed-phase HPLC was strongly dependent on the percentage and strength of the organic solvent used in the mobile phase [16,17,18]. In this study, methanol was selected over acetonitrile as it provided better peak shapes, especially for fangchinoline and tetrandrine.

To obtain good sensitivity and accuracy for the simultaneous quantification of the marker compounds, the DAD detection wavelength was set to 235 nm, which was a compromise between the UV absorption maxima of the marker compounds and interfering components in the extract. Measurement at 235 nm exhibited sufficient sensitivity and a stable chromatographic baseline.

**Figure 2:**
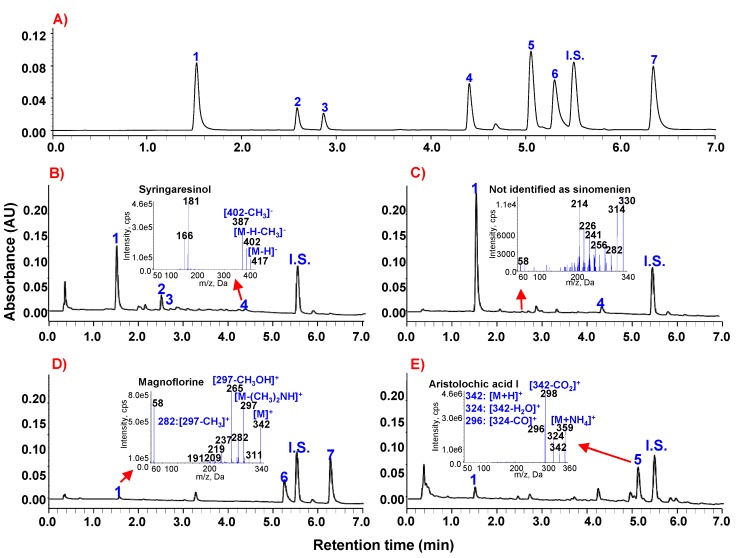
Representative UHPLC chromatograms of marker compounds: (**A**) standard mixtures (**B**) *Sinomenium acutum*; (**C**) *Cocculus trilobus*; (**D**) *Stephania tetrandra*; and (**E**) *Aristolochia fangchi*. Peak identities: 1: magnoflorine; 2: sinomenine; 3: isosinomenine; 4: syringaresinol; 5: aristolochic acid І; 6: fangchinoline; 7: tetrandrine; and I.S.: propyl-4-hydroxybenzoate.

The UHPLC-DAD analysis conditions that produced the best chromatographic separation and detection sensitivities of the seven markers were determined to be a solvent system of 20 mM NH_4_OAc (adjusted to pH 6.0 with acetic acid)–methanol and detection wavelength at 235 nm. Using the established UHPLC method, diverse marker compounds were successfully separated within a 7 min run time and had resolutions of greater than 2.60, even for the closest peaks, which were aristolochic acid I and fangchinoline (Figure 2A). This UHPLC-DAD method was also successfully applied to determine the level of marker compounds in four typical Fangchi extracts, as shown in Figure 2B~E. Identification of the analytes is based on reference compounds and MS/MS data (see insets of Figure 2B~E). UHPLC chromatograms of extracts of Fangchi species exhibited stable baselines and did not show any significant interferences.

### 2.2. UHPLC-ESI-MS/MS Confirmation of UHPLC-DAD Results

**Figure 3:**
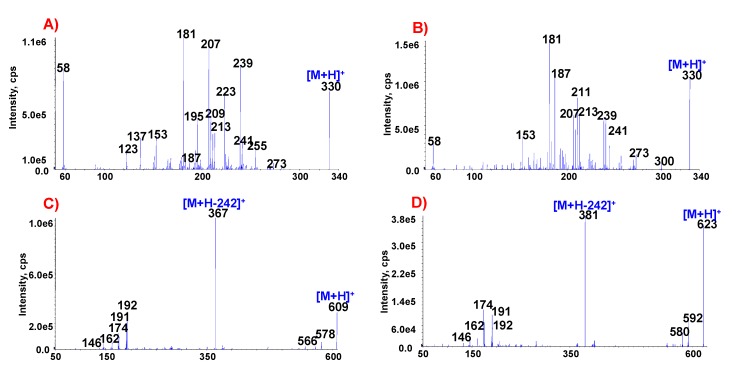
ESI-MS/MS spectra of marker compounds found in different Fangchi species. (**A**) sinomenine; (**B**) isosinomenine; (**C**) fangchinoline; (**D**) tetrandrine.

In some cases, it is difficult to accurately identify markers in herbal extracts by LC-DAD due to the presence of interfering compounds with structures that are similar to the markers. For the accurate identification of various types of markers extracted from Fangchi species, authentic markers were preferentially analyzed by ESI-MS/MS to determine the specificity of MS/MS spectral patterns for different molecules. The MS/MS spectra of the seven marker compounds are shown in the insets of Figure 2 and Figure 3. The MS/MS spectral data of the authentic standards are summarized in Table 1, including exact mass measurements of precursor ions and characteristic fragment ions. The exact mass measurements of the markers are indistinguishable from their theoretical values and are within 2.6 mmu of error. The MS/MS spectra of each of the markers exhibited specific patterns.

**Scheme 1:**
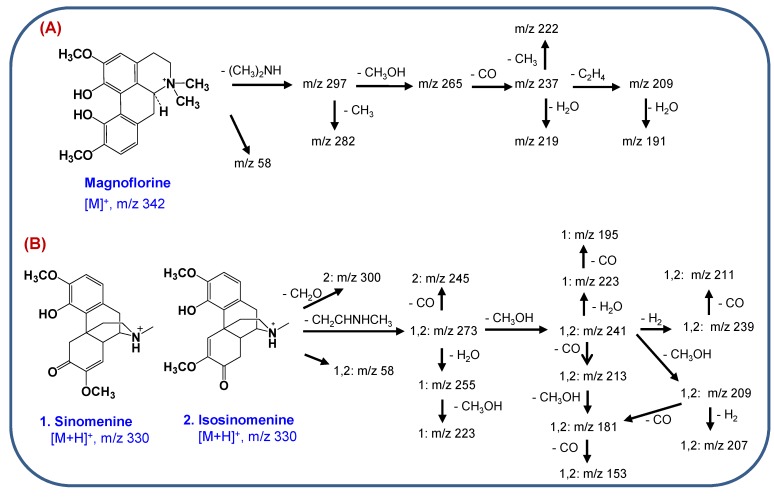
Proposed MS/MS fragmentation pathways of (**A**) magnoflorine and (**B**) the morphine alkaloids.

Magnoflorine, an aporphine alkaloid, showed a molecular ion [M]^+^ at *m/z* 342 and the initial fragmentation was due to the loss of dimethylamine [(CH_3_)_2_NH]^+^, as shown in the inset of Figure 2C. Other characteristic ions were produced by the successive losses of substituted CH_3_ groups or CH_3_OH molecules from the [M-(CH_3_)_2_NH]^+^ ion [19,20]. The proposed MS/MS fragmentation pathways for magnoflorine are shown in Scheme 1A.

The MS/MS spectra of sinomenine and isosinomenine, which are structural isomers, exhibited different spectral patterns due to the different positions of the double bonds in the C-ring, as shown in Figure 3A,B. The MS/MS spectral patterns of these isomers can be characterized by the presence of various fragment ions due mainly to cleavage of the piperidine ring. Initial MS fragmentations are a loss of an amine moiety (CH_2_CHNHCH_3_) from [M+H]^+^ induced by successive cleavage of the piperidine ring [21]. The other characteristic fragment ions at *m/z* 181, 207 and 58 were formed by losses of CH_3_OH and CO molecules from the [(M+H)-CH_2_CHNHCH_3_]^+^ ion [21]. MS/MS fragmentation pathways of the morphine alkaloids are proposed in Scheme 1B.

**Table 1:** Exact mass measurements of precursor ions (*m/z*) and characteristic fragment ions (*m/z*) for seven marker compounds.

Compound	Precursor ion formula	Precursor ion (*m/z*)	Exact mass (*m/z*)			Characteristic ions (*m/z*)
			Theoretical	Observed ^a^	Difference (mmu)	
Magnoflorine	C_20_H_24_NO_4_^+^	342	342.1705	342.1724	1.9	58, 191, 209, 219, 237, 265, 282, 297, 311
Sinomenine	C_19_H_24_NO_4_^+^	330	330.1705	330.1716	1.1	58, 123, 137, 153, 181, 187, 195, 207, 209, 213, 223, 239, 241, 255, 273
Isosinomenine	C_19_H_24_NO_4_^+^	330	330.1705	330.1721	1.6	58, 153, 181, 187, 207, 211, 213, 239, 241, 273, 300
Syringaresinol	C_22_H_25_O_8_^−^	417	417.1549	417.1545	0.4	166, 181, 387, 402
Fangchinoline	C_37_H_41_N_2_O_6_^+^	609	609.2965	609.2991	2.6	146, 162, 174, 191, 192, 367, 566, 578
Aristolochic acid I	C_17_H_15_N_2_O_7_^+^	359	359.0879	359.0884	0.5	296, 298, 324, 342
Tetrandrine	C_38_H_43_N_2_O_6_^+^	623	623.3121	623.3142	2.1	146, 162, 174, 191, 192, 381, 580, 592

^a^ obtained by high-resolution fast atom bombardment mass spectrometry.

**Scheme 2:**
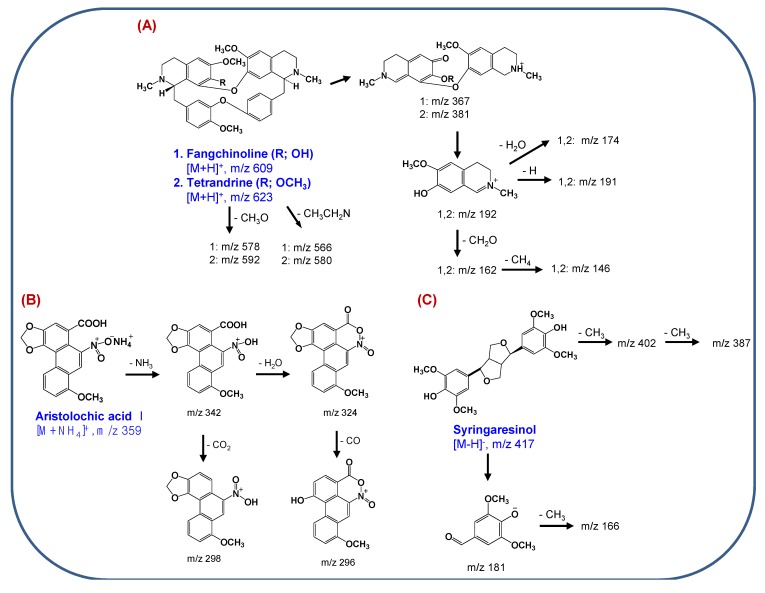
Proposed MS/MS fragmentation pathways of (**A**) bisbenzylisoquinoline alkaloids, (**B**) aristolochic acid I and (**C**) syringaresinol.

The MS/MS spectra of the bisbenzylisoquinoline alkaloids exhibited [M+H]^+^ ions and characteristic diagnostic ions at *m/z* 367 and 381 for fangchinoline and tetrandrine, respectively, due to the preferred cleavage of the carbon-carbon bond that is β both to the nitrogen and to the two aromatic systems, followed by *O*-demethylation of an aromatic methoxyl group (Figure 3C,D). The product ion at *m/z* 192 most likely corresponds to a single isoquinoline moiety that can lose a H_2_O molecule, resulting in the formation of an ion at *m/z* 174 [22]. MS/MS fragmentation pathways of the bisbenzylisoquinoline alkaloids are suggested in Scheme 2A.

The ESI-MS spectrum of aristolochic acid I exhibited an ammoniated ion [M+NH_4_]^+^ at *m/z* 359, due to the easy formation of an adduct of an ammonium ion with the nitro group in positive ion mode. The ammonium is derived from the ammonium acetate in the mobile phase. As shown in Figure 2D, specific product ions at *m/z* 342 and 298 resulting from successive losses of NH_3_ and CO_2_ from the parent [(M+NH_4_)]^+^ ion were observed. Two additional specific fragment ions (*m/z* 324 and *m/z* 296) were observed, which correspond to [(M+NH_4_)-NH_3_-H_2_O]^+^ and [[(M+NH_4_)-NH_3_-H_2_O-CO]^+^ ions. Notably, the loss of a H_2_O molecule from [(M+NH_4_)-NH_3_]^+^ ion might induce cyclization between the carboxylic acid and nitro groups [23,24]. The MS/MS fragmentation pathways of aristolochic acid I are proposed in Scheme 2B.

Syringaresinol, a furofuranolignan containing two phenol groups, was observed with more sensitivity in negative-ion mode ESI-MS than in positive-ion mode, producing an [M−H]^−^ ion (*m/z* 417) (inset of Figure 2A). The collision-induced dissociation (CID) spectrum of syringaresinol contained characteristic fragment ions at *m/z* 402 and *m/z* 387, resulting from the successive losses of two CH_3_ groups from the [M−H]^−^ ion [25]. The most abundant ion at *m/z* 181 might be formed by cleavage of a tetrahydrofuran ring, as shown in Scheme 2C. 

Based on these results, the presence of the marker compounds in extracts of Fangchi species was further confirmed, although the marker compound peaks were small. As shown Figure 2B, the small peak at 2.3 min in the *C. trilobus* extract was erroneously identified as sinomenine based on the UHPLC retention time and ESI-MS results. However, based on its MS/MS spectrum, it was determined that the peak was not sinomenine. The peak at 2.3 min is hypothesized to be a morphine alkaloid based on its MS/MS spectral pattern (inset of Figure 2C) and retention time. At the present time, the identity of this unknown compound is ambiguous and warrants further investigation. 

All herbal extracts were analyzed using the UHPLC-MS/MS system to accurately identify markers compounds. Magnoflorine and syringaresinol were observed for the first time in *S. tetrandra* and *C. trilobus*, respectively, in this study. The MS/MS fragmentation patterns of these markers are useful for identifying other bioactive components and related compounds in Fangchi species and other herbal medicines.

### 2.3. Method Validation

The sample used for studies to determine the recovery and precision of the method was an *S. acutum* extract containing measurable levels of the markers compounds. Calibration curves were constructed in the concentration range of 0.2–100 μg/mL for each of the markers. Each of the seven standard solutions was analyzed in triplicate. Multi-point calibration curves were constructed by linear regression analysis of the peak ratios of each analyte to the internal standard, *versus* concentration. The calibration equations, linear correlation coefficients, limit of detection (LOD) and limit of quantitation (LOQ) of markers are summarized in Table 2. The correlation coefficients were all greater than 0.9981, indicating good linearity. Calibration curves prepared in the absence and presence of the sample matrix were almost identical within experimental error. Calibration in the absence of the sample matrix is recommended for screening because it is simpler than calibration in the presence of the matrix [26,27]. The LODs and LOQs of the markers were measured as the lowest concentrations that corresponded to signal-to-noise ratios of 3 and 10, respectively. The LODs and LOQs of the marker compounds were in the ranges of 0.01–0.05 μg/mL and 0.05–0.2 μg/mL, respectively. 

To test the accuracy and precision of the analytical method, known amounts of the seven markers (5, 25 and 50 μg/mL) were added to *S. acutum* extract. The intra- and inter-day variations for the seven marker compounds in the *S. acutum* extract were determined as described in the Experimental section, and the results are summarized in Table 3. The precision of the method for the simultaneous determination of the seven marker compounds was acceptable because the RSD did not exceed 7.78% at concentrations of 5, 25 and 50 μg/mL. At the same concentrations, the intra-day accuracies ranged from 94.49% to 100.84%, while the inter-day accuracies ranged from 93.81% to 100.83%, indicating good accuracy of the method. These good results indicate that the marker compounds were completely extracted from the Fangchi sample by sonication extraction using 70% MeOH. The reproducibility of retention times was evaluated over 36 injections using the standard solution, and the RSD was 0.14%–1.18%. The repeatability of the area ratio was calibrated using the peak area of each compound divided by the peak area of the internal standard, and the RSD was 1.62%–3.85% (n = 9). These results indicated that the UHPLC method had suitable repeatability with respect to both retention time and peak area.

**Table 2:** Calibration curve equations, linearity correlation coefficients, LOD values and LOQ values for the marker compounds.

Compound	Range (μg/mL)	Linear equation	Correlation coefficient	LOD (μg/mL)	LOQ (μg/mL)
Magnoflorine	0.2–100	y = 0.0402x − 0.0161	0.9990	0.01	0.05
Sinomenine	0.2–100	y = 0.0090x − 0.0024	0.9989	0.05	0.17
Isosinomenine	0.2–100	y = 0.0056x − 0.0036	0.9995	0.05	0.20
Syringaresinol	0.2–100	y = 0.0221x + 0.0021	0.9981	0.03	0.10
Fangchinoline	0.2–100	y = 0.0885x − 0.0411	0.9998	0.03	0.10
Aristolochic acid І	0.2–100	y = 0.0311x − 0.014	0.9982	0.01	0.05
Tetrandrine	0.2–100	y = 0.0511x + 0.0786	0.9982	0.02	0.07

**Table 3:** Intra- and inter-day precision and accuracy for the quantification of marker compounds in spiked *S. acutum* extracts.

Compound	Fortified conc. (μg/mL)	Intra-day (n = 5)				Inter-day (n = 5)		
		Observed Conc. (μg/mL)	Precision (%)	Accuracy (%)		Observed Conc. (μg/mL)	Precision (%)	Accuracy (%)
Magnoflorine	5	4.72	5.19	94.49		4.69	5.27	93.81
	25	24.78	0.63	99.14		24.69	2.22	98.78
	50	49.13	1.37	98.26		49.45	2.97	98.90
Sinomenine	5	4.90	6.54	98.05		4.97	4.10	99.49
	25	24.81	1.68	99.23		25.19	3.97	100.76
	50	48.35	0.11	96.70		48.66	1.25	97.31
Isosinomenine	5	5.04	7.09	100.84		4.98	4.34	99.63
	25	23.86	7.78	95.46		24.53	5.16	98.11
	50	49.93	1.44	99.86		50.27	3.08	100.53
Syringaresinol	5	4.80	4.03	96.02		4.86	1.99	97.15
	25	24.74	0.16	98.95		24.75	1.82	98.99
	50	49.87	0.66	99.75		49.56	1.30	99.11
Fangchinoline	5	4.80	2.83	95.97		4.74	2.12	94.84
	25	24.53	5.02	98.11		24.54	2.09	98.17
	50	50.36	0.15	100.72		48.79	4.67	97.58
Aristolochic acid I	5	4.96	6.15	99.26		5.04	3.66	100.83
	25	24.82	0.62	99.29		25.12	1.36	100.47
	50	50.41	0.50	100.82		49.29	4.69	98.52
Tetrandrine	5	4.85	0.66	97.08		4.97	3.37	99.48
	25	24.55	1.59	98.18		24.72	2.44	98.86
	50	50.36	0.27	100.72		49.58	2.67	99.16

### 2.4. Method Application

Twenty Fangchi samples of different origins (*A. fangchi*, *S. acutum*, *S. tetrandra* and *C. trilobus*) were successfully analyzed using the established method for quality control purposes. Typical UHPLC chromatograms of extracts from four Fangchi species that showed different patterns based on their origins are shown in Figure 2. As shown in this Figure, the established method was successfully applied for the determination of marker compounds in different Fangchi species and there were substantial differences in the constituents of the samples from different species. 

**Figure 4:**
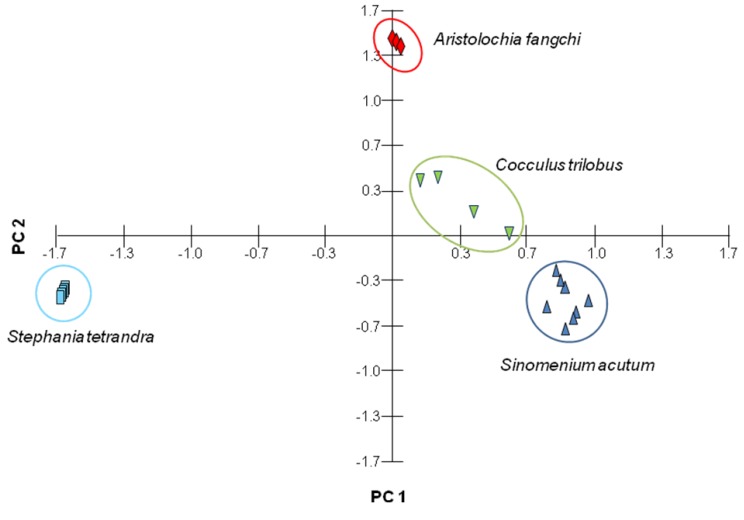
PCA plot of different Fangchi species combined with 20 chromatographic data.

The amounts of each compound in the various Fangchi species extracts are listed in Table 4. Seven compounds were observed with markedly different abundances among the Fangchi species under our experimental conditions. The RSDs for the marker compounds ranged from 0.02%–5.91%. Magnoflorine was observed in all samples, but the amount varied significantly depending on the sample origin. In this study, magnoflorine was observed for the first time in *S. tetrandra*, as shown in the inset of Figure 2D. The morphine alkaloids were observed in all of the *S. acutum* samples and the bisbenzylisoquinolines were observed in all *S. tetrandra* samples. The lignan, syringaresinol, was detected as a minor component of the *S. acutum* and *C. trilobus* samples. Aristolochic acid I could prove to be an important marker, although it was only observed in small quantities in *A. fangchi*. These results indicate UHPLC-DAD is a useful tool for the determination of the seven characteristic marker compounds used for the quality control of four Fangchi species and for classification of the origins of herbal material by PCA (Figure 4).

**Table 4:** The amounts of marker compounds in extracts of Fangchi species of different origins.

Sample	Mean concentration (mg/g) ± standard deviation (relative standard deviation)						
	Magnoflorine	Sinomenine	Isosinomenine	Syringaresinol	Fangchinoline	Tetrandrine	Aristolochic acid І
*S. acutum* 1	21.08 ± 0.03 (0.03)	1.70 ± 0.26 (3.10)	2.35 ± 0.27 (2.27)	2.44 ± 0.07 (0.61)	-	-	-
*S. acutum* 2	9.61 ± 0.01 (0.03)	1.69 ± 0.21 (2.44)	1.34 ± 0.20 (2.95)	0.24 ± 0.01 (1.22)	-	-	-
*S. acutum* 3	7.46 ± 0.02 (0.05)	3.68 ± 0.03 (0.16)	1.73 ± 0.02 (0.27)	0.64 ± 0.02 (0.76)	-	-	-
*S. acutum* 4	8.22 ± 2.07 (5.04)	3.79 ± 1.12 (5.91)	0.30 ± 0.02 (1.48)	0.38 ± 0.09 (4.90)	-	-	-
*S. acutum* 5	8.53 ± 0.55 (1.30)	6.30 ± 0.51 (1.62)	3.02 ± 0.24 (1.59)	0.08 ± 0.01 (3.23)	-	-	-
*S. acutum* 6	25.06 ± 0.03 (0.03)	24.35 ± 0.02 (0.02)	14.10 ± 0.05 (0.07)	0.14 ± 0.02 (2.90)	-	-	-
*S. acutum* 7	6.65 ± 0.03 (0.10)	13.28 ± 0.04 (0.06)	4.86 ± 0.01 (0.04)	0.02 ± 0.01 (5.75)	-	-	-
*S. acutum* 8	5.69 ± 1.14 (4.02)	11.03 ± 0.83 (1.50)	0.55 ± 0.10 (3.65)	0.03 ± 0.004 (2.57)	-	-	-
*C. trilobus* 1	9.38 ± 0.78 (1.67)	-	-	0.51 ± 0.10 (3.82)	-	-	-
*C. trilobus* 2	17.21 ± 0.04 (0.04)	-	-	0.01 ± 0.001 (2.90)	-	-	-
*C. trilobus* 3	3.14 ± 0.22 (1.40)	-	-	0.21 ± 0.05 (4.32)	-	-	-
*C. trilobus* 4	18.85 ± 0.03 (0.03)	-	-	0.31 ± 0.02 (1.27)	-	-	-
*S. tetrandra* 1	0.14 ± 0.01 (0.78)	-	-	-	3.14 ± 0.01 (0.07)	2.80 ± 0.02 (0.12)	-
*S. tetrandra* 2	0.37 ± 0.01 (0.50)	-	-	-	5.49 ± 0.01 (0.05)	6.35 ± 0.02 (0.06)	-
*S. tetrandra* 3	0.33 ± 0.06 (3.83)	-	-	-	5.26 ± 0.57 (2.16)	5.86 ± 1.13 (3.87)	-
*S. tetrandra* 4	0.58 ± 0.02 (0.60)	-	-	-	8.08 ± 0.02 (0.04)	10.31 ± 0.02 (0.05)	-
*S. tetrandra* 5	0.37 ± 0.03 (1.73)	-	-	-	6.41 ± 0.64 (1.99)	9.31 ± 0.41 (0.89)	-
*A. fangchi* 1	0.17 ± 0.004 (0.47)	-	-	-	-	-	0.10 ± 0.001 (0.28)
*A. fangchi* 2	0.27 ± 0.01 (0.38)	-	-	-	-	-	0.40 ± 0.01 (0.62)
*A. fangchi* 3	0.20 ± 0.01 (0.52)	-	-	-	-	-	0.20 ± 0.003 (0.29)

## 3. Experimental

### 3.1. Materials and Reagents

All reagents and organic solvents were analytical grade. Acetonitrile, methanol, ethanol and acetone were purchased from J.T. Baker (Phillipsburg, NJ, USA). Sinomenine, isosinomenine, magnoflorine and syringaresinol were isolated from Fangchi species using a previously reported method [28]. The purity and identity of these compounds were determined by HPLC-DAD and several spectroscopic methods. Tetrandrine (purity ≥ 90%) was purchased from Sigma-Aldrich (Milwaukee, WI, USA). Fangchinoline and Aristolochic acid І (purity > 98%) were purchased from Chengdu Biopurify Phytochemicals Ltd. (Sichuan, China). Propyl-4-hydroxybenzoate, which was used as an internal standard (I.S.), was purchased from Daejung (Eumseong, Korea, purity > 99%). All standards were kept in a refrigerator. The chemical structures of the standards used in this study are depicted in Figure 1. Samples of Fangchi species of different origins were collected from herbal markets in Korea and China.

### 3.2. Preparation of Standards

Stock solutions were prepared by dissolving 1 mg of each standard (sinomenine, isosinomenine, magnoflorine, tetrandrine, fangchinoline and syringaresinol) in 1 mL of methanol, and 1 mg of aristolochic acid І in 1 mL of acetone. Each stock solution was diluted to create seven calibration points (0.2, 0.5, 1, 5, 10, 50 and 100 µg/mL) for preparation of the calibration curves. The concentration of propyl-4-hydroxybenzoate (I.S.) was 100 µg/mL for all analytes. All stock solutions were stored at −4 °C until analysis. 

### 3.3. Preparation of Crude Rug Extracts

Dried Fangchi species were pulverized and the resulting powders were screened through 30 mesh sieves. One gram of powder was placed into 20 mL of 70% methanol. The sample mixture was extracted for 30 min in 42 KHz of an ultrasonic bath (Branson 5510, Branson Ultrasonic Corp., Danbury, CT, USA) at room temperature. After extraction, the sample mixture was centrifuged twice at 3,000 rpm for 10 min. The supernatant was collected and filtered through a 0.22 µm membrane filter (GHP membrane filters, 0.20 μm pore size, Woongki Science, Seoul, Korea), and propyl-4-hydroxybenzoate (I.S.) was added to the extract solution to obtain a final solution of 5 mg powder/mL for, *S. acutum*, *S. tetrandra* and *C. trilobus*, 25 mg powder/mL *A. fangchi*, and 100 µg/mL propyl-4-hydroxybenzoate prior to injection into the UHPLC system. Different extraction methods, including classical ultra-sonication, reflux and immersion, were tested. 

### 3.4. UHPLC-DAD Conditions

UHPLC analysis was performed using a WATERS Acquity UPLC system (Waters, Milford, MA) equipped with a quaternary solvent delivery manager, a column manager, a sample manager and a diode array detector (DAD). The chromatographic separation analysis was carried out on a WATERS (Milford, MA, USA) Acquity UPLC ® BEH C18 column (50 × 2.1 mm, i.d., 1.7 µm) connected to an Acquity UPLC ® BEH C18 VanGuard™ pre-column (5 × 2.1 mm, i.d., 1.7 µm). The mobile phases consisted of solvent A (20 mM NH_4_OAc at pH 6.0, adjusted with acetic acid) and solvent B (methanol). The gradient elution mode was programmed as follows: 10%–50% B for 0.0–3.5 min and 50%–80% B for 3.5–7.0 min. The UV detection wavelength was 235 nm. The flow rate and injection volume were 0.4 mL/min and 0.5 µL, respectively.

### 3.5. UHPLC-ESI-MS Conditions

All ESI-MS experiments were performed using an API 3200 instrument (MDS Sciex, Concord, ON, Canada) equipped with an ESI source. The chromatographic conditions were the same as those used for UHPLC-DAD with the exception of the NH_4_OAc concentration. To avoid damage to the MS system, 15 mM NH_4_OAc at pH 6.0 (adjusted with acetic acid) was used as solvent A. All MS parameters were optimized according to the manufacturer’s instructions. In positive-ion mode ESI experiments, the mass spectrometric conditions were as follows: curtain gas, 20 psi; electron voltage, 4500 V; temperature, 400 °C; nebulizing gas, 50 psi; and heating gas, 50 psi; the mass scan range was *m/z* 50–635 in the full-mass scan. The collision energy was 40% in Q2. A syringe pump was used for direct analysis of the seven reference compounds at a flow rate of 10 µL/min. The ESI-MS/MS experimental conditions were as follows: magnoflorine, declustering potential (DP) 50 V and collision energy (CE) 35%; sinomenine and isosinomenine, DP 50 V and CE 44%; aristolochic acid, DP 25 V and CE 15%; fangchinoline and tetrandrine, DP 81 V and CE 55%. In the negative-ion mode ESI experiments, the mass spectrometric conditions were as follows: curtain gas, 10 psi; electron voltage, −4,500 V; temperature, 400 °C; nebulizing gas, 50 psi; and heating gas, 50 psi; the mass scan range was *m/z* 50–430 in the full-mass scan. The ESI-MS/MS experimental conditions for syringaresinol were DP −35 V and CE 30%. 

### 3.6. Method Validation

Validation of the analytical method for the seven marker compounds was determined by the linearity, LOD, LOQ, accuracy and precision. The linear calibration curves were performed at least five times for each reference compound and were constructed by plotting the ratios of peak areas (analytes/internal standard) against the concentrations for each compound. Precision, accuracy and intra- and inter-day recovery tests were performed by adding known amounts of standards (5, 25 and 50 µg/mL) to *S. acutum* (n = 5). Precision and accuracy were expressed as relative standard deviations (RSDs) and recoveries (%), respectively. 

## 4. Conclusions

In this study, a rapid and simple UHPLC-DAD method was developed and validated for the simultaneous determination of syringaresinol, aristolochic acid І and five alkaloids in four Fangchi species of different origins. These marker compounds, comprising morphine, aporphine and bisbenzylisoquinoline alkaloids, a nitrophenanthrene carboxylic acid and a lignan were successfully identified by UHPLC-ESI-MS/MS in four Fangchi species extracts, providing confirmation of the UHPLC-DAD results. Furthermore, in this study, magnoflorine and syringaresinol were observed for the first time in *S. tetrandra* and *C. trilobus*, respectively. Four different Fangchi species could be readily distinguished from one another based on the presence and levels of specific marker compounds. UHPLC-DAD combined with MS/MS analysis was found to be a useful and practical tool for the quality control of Fangchi species.
